# Editorial: Flourishing in arid realms: exploring the adaptation of plant functional traits to drought environments

**DOI:** 10.3389/fpls.2025.1627814

**Published:** 2025-06-02

**Authors:** Jie Gao, Kyung-Min Kim, Johan Gielis, Weiguo Liu

**Affiliations:** ^1^ College of Life Sciences, Xinjiang Normal University, Urumqi, China; ^2^ Institute of Coastal Agriculture, Kyungpook National University, Daegu, Republic of Korea; ^3^ Department of Biosciences Engineering, University of Antwerp, Antwerp, Belgium; ^4^ College of Ecology and Environment, Xinjiang University, Urumqi, China

**Keywords:** plant functional traits, global climate change, drought resistance, molecular mechanisms, plant-soil microbial interactions

In the context of global climate change, the increasing frequency and intensity of droughts pose significant threats to plant growth and ecosystem stability. These challenges highlight the urgent need to understand how plants adapt to water scarcity across molecular, physiological, and ecological scales. Plant functional traits—morphological, physiological, and phenological characteristics that influence plant performance—have emerged as key indicators in studying plant strategies for drought resistance. Understanding how plants utilize their functional traits to survive, reproduce, and maintain community structure and ecological functions under drought stress has become a central issue in ecology and plant physiology.

This Research Topic, titled “Flourishing in Arid Realms,” comprises 18 articles focusing on the regulation of plant functional traits and environmental adaptation mechanisms under drought stress. The contributing studies offer diverse perspectives and span multiple biological levels, highlighting recent progress and critical gaps in our understanding of drought adaptation. The Research Topic encompasses various levels, from gene regulation, morphological structures, physiological metabolism, to community responses and ecosystem functions, providing substantial empirical support and theoretical insights into plant drought resistance mechanisms.

Regarding the molecular mechanisms of plant drought resistance, a study on the AmMADS47 gene from Artemisia mongolica demonstrated that its heterologous expression in rice negatively regulates drought tolerance (Fan et al.). This finding reveals the complex functions of MADS-box family genes in drought response and offers potential targets for crop drought-resistant breeding. Similarly, interspecific hybrid rootstocks of two drought-tolerant pistachio species exhibited significantly different physiological and molecular responses under drought stress, highlighting the importance of genetic background and interspecific interactions in forming drought resistance (Osku et al.). These results suggest that even with similar phenotypic drought tolerance, there may be significant differences in molecular regulatory pathways, necessitating more detailed analyses.

From the perspective of plant-soil microbial interactions, arbuscular mycorrhizal colonization significantly influences root structure and resource acquisition strategies. Research indicates that in extreme drought environments, arbuscular mycorrhizal colonization can define plant root ecological strategies, effectively enhancing water and nutrient uptake and optimizing rhizosphere resource allocation (Delpiano et al.). Roots, as the frontline of plant drought resistance, exhibit notable changes in functional traits under drought stress. For instance, in cotton, drought stimulates increased exudation of organic nitrogen from roots, which may serve as an adaptive metabolic response and potentially alter rhizosphere microbial community structures, further affecting overall plant drought performance (Coker et al.).

Environmental factors regulate plant traits differently across various geographical and ecological conditions. Studies in central Yunnan grasslands show that slope aspect and elevation jointly influence interspecific relationships within herbaceous plant communities, revealing the role of microtopography in shaping community structure and species interactions (Gong and Gong). In the desert regions of northwest China, Larix species in dune areas exhibit varying water use strategies along precipitation gradients, reflecting plants’ high sensitivity to changes in water availability (Xu et al.). Such studies aid in identifying typical plant strategies adapted to arid habitats and provide theoretical foundations for managing drought ecosystems and vegetation restoration.

Comparative studies between desert herbaceous and shrub species reveal significant differences in the coordination patterns and variability of root and leaf traits. Herbaceous plants display stronger trait coupling, while shrubs exhibit greater plasticity in specific traits (Ma et al.). This differentiation reflects the functional strategy divergence formed by different plant life forms during long-term drought adaptation. Additionally, research indicates that along elevation gradients, leaf nutrient traits exhibit greater environmental plasticity than resource utilization traits, suggesting that nutrient acquisition strategies respond more rapidly to climate changes (Zhang et al.).

Under human intervention, various soil and water conservation measures significantly impact plant functional traits. In the Loess Plateau, implementing multiple conservation strategies alters vegetation structure and functional trait configurations, indicating that human activities can influence and guide plant adaptation pathways to drought stress (Duan et al.). In California’s arid shrub ecosystems, Ephedra species modify local microclimates, indirectly affecting their own and neighboring plants’ survival environments, demonstrating plants’ ability to reshape environmental conditions through structural functions (Ghazian et al.).

Drought stress profoundly affects plant productivity and physiological metabolism. Studies in northwest China show that four introduced Ranunculus species exhibit differences in organ nutrient allocation during various reproductive stages, reflecting adaptive adjustments in reproductive energy allocation strategies (Liu et al.). Modern bread wheat under drought stress shows reduced β-diketone accumulation, indicating that intense stress may disrupt stable metabolic regulatory networks, affecting crop quality (Kuruparan et al.). Meanwhile, in semi-arid grasslands, nitrogen addition and mixed sowing of Bothriochloa ischaemum and Lespedeza davurica significantly influence plant chlorophyll fluorescence characteristics and community productivity, demonstrating the adjustability of community functions under external resource inputs (Wang et al.).

Precise quantification and modeling of functional traits are fundamental to understanding plant ecological strategies. A study on the leaf area distribution of Alangium chinense compared five mathematical equations to assess their adaptability in describing variations in leaf shape, contributing to the construction of a universal trait prediction framework (Deng et al.). Regarding leaf trait correlations, research on Red Tip and Chinese photinias revealed a significant positive correlation between leaf vein length per unit area and stomatal density, providing direct evidence of the functional link between leaf structure and water-use efficiency (He et al.).

It is worth noting that differences in forest origin—namely, between planted and natural forests—have a significant influence on the strategies plants adopt for resource use and allocation (Gong et al., 2023). Numerous studies have demonstrated that planted and natural forests differ markedly in their responses of plant functional traits to environmental conditions. In particular, natural forests tend to exhibit greater environmental plasticity, allowing them to maintain higher stability and adaptability under complex or changing ecological scenarios (Gao et al., 2023). This heightened plasticity may help explain why functional traits in natural forests often outperform those in planted ones. In contrast, functional traits in planted forests show a stronger response to environmental changes, indicating a higher degree of sensitivity and potentially weaker ecological adaptability (Zhang et al.). These findings highlight the importance of carefully considering the adaptive capacities of plant materials when planning ecological restoration and vegetation recovery, as doing so is critical for enhancing the long-term stability and resilience of restored ecosystems.

Overall, the 18 articles included in this Research Topic reveal various plant adaptation strategies under drought stress, demonstrating the potential for multi-scale integration in functional trait research. Whether in gene expression regulation, metabolic responses, trait variation patterns, or community dynamics, this Research Topic provides significant theoretical and empirical support. These findings not only deepen our understanding of how plants respond to water-limited environments but also help build a more predictive framework for plant behavior under future climate scenarios. We believe these research findings not only enrich the knowledge system of drought ecology but also offer a solid scientific basis for future vegetation restoration, ecosystem management, and sustainable development of agriculture and forestry under global change. The integration of such trait-based approaches into policy-making and land-use planning will be essential to build resilient ecosystems that can withstand the growing challenges posed by climate change.

## References

[B1] Gao J. Ji Y. Zhang X. (2023). Net primary productivity exhibits a stronger climatic response in planted versus natural forests. For. Ecol. Manage. 529, 120722. doi: 10.1016/j.foreco.2022.120722

[B2] Gong H. Song W. Wang J. Wang X. Ji Y. Zhang X.. (2023). Climate factors affect forest biomass allocation by altering soil nutrient availability and leaf traits. J. Integr. Plant Biol. 65, 2292–2303. doi: 10.1111/jipb.13545 37470341

